# Traditional and Emerging Lifestyle Risk Behaviors and All-Cause Mortality in Middle-Aged and Older Adults: Evidence from a Large Population-Based Australian Cohort

**DOI:** 10.1371/journal.pmed.1001917

**Published:** 2015-12-08

**Authors:** Ding Ding, Kris Rogers, Hidde van der Ploeg, Emmanuel Stamatakis, Adrian E. Bauman

**Affiliations:** 1 Prevention Research Collaboration, Sydney School of Public Health, University of Sydney, Camperdown, New South Wales, Australia; 2 Charles Perkins Centre, University of Sydney, Camperdown, New South Wales, Australia; 3 George Institute for Global Health, Sydney, New South Wales, Australia; 4 Department of Public and Occupational Health, EMGO Institute for Health and Care Research, VU University Medical Centre, Amsterdam, the Netherlands; 5 Exercise and Sports Science, Faculty of Health Sciences, University of Sydney, Camperdown, New South Wales, Australia; Makerere University Medical School, UGANDA

## Abstract

**Background:**

Lifestyle risk behaviors are responsible for a large proportion of disease burden worldwide. Behavioral risk factors, such as smoking, poor diet, and physical inactivity, tend to cluster within populations and may have synergistic effects on health. As evidence continues to accumulate on emerging lifestyle risk factors, such as prolonged sitting and unhealthy sleep patterns, incorporating these new risk factors will provide clinically relevant information on combinations of lifestyle risk factors.

**Methods and Findings:**

Using data from a large Australian cohort of middle-aged and older adults, this is the first study to our knowledge to examine a lifestyle risk index incorporating sedentary behavior and sleep in relation to all-cause mortality. Baseline data (February 2006– April 2009) were linked to mortality registration data until June 15, 2014. Smoking, high alcohol intake, poor diet, physical inactivity, prolonged sitting, and unhealthy (short/long) sleep duration were measured by questionnaires and summed into an index score. Cox proportional hazards analysis was used with the index score and each unique risk combination as exposure variables, adjusted for socio-demographic characteristics.

During 6 y of follow-up of 231,048 participants for 1,409,591 person-years, 15,635 deaths were registered. Of all participants, 31.2%, 36.9%, 21.4%, and 10.6% reported 0, 1, 2, and 3+ risk factors, respectively. There was a strong relationship between the lifestyle risk index score and all-cause mortality. The index score had good predictive validity (*c* index = 0.763), and the partial population attributable risk was 31.3%. Out of all 96 possible risk combinations, the 30 most commonly occurring combinations accounted for more than 90% of the participants. Among those, combinations involving physical inactivity, prolonged sitting, and/or long sleep duration and combinations involving smoking and high alcohol intake had the strongest associations with all-cause mortality. Limitations of the study include self-reported and under-specified measures, dichotomized risk scores, lack of long-term patterns of lifestyle behaviors, and lack of cause-specific mortality data.

**Conclusions:**

Adherence to healthy lifestyle behaviors could reduce the risk for death from all causes. Specific combinations of lifestyle risk behaviors may be more harmful than others, suggesting synergistic relationships among risk factors.

## Abstract 2

Analysis of large population cohort reveals short and long sleep duration and extended sitting for sedentary workers as lifestyle risks that can be added to other known mortality risk factors.

## Editors' Summary

**Background:**

For the first time in human history, noncommunicable diseases (NCDs) are killing more people than infectious diseases. Every year, about 38 million people die from a NCD—more than two-thirds of the world’s annual deaths. The most common NCDs are cardiovascular diseases (conditions that affect the heart and the circulation), cancers, diabetes, and chronic respiratory diseases (long-term diseases that affect the lungs and airways). These NCDs can be largely attributed to modifiable lifestyle risk factors such as smoking, harmful use of alcohol, physical inactivity, and having an unhealthy diet (one with low fruit and vegetable intake and high saturated fat and salt intake). More recently, a sedentary lifestyle (sitting for more than seven hours during a typical 24-hour day—separate from whether a person is physically active, i.e., undertaking more than 150 minutes of moderate-to-vigorous physical activity every week) and having an unhealthy sleep pattern (less than seven hours or more than nine hours of sleep per day) have also been identified as modifiable risk factors for NCDs.

**Why Was This Study Done?:**

It should be possible to reduce the burden of NCDs by encouraging people to adopt a healthier lifestyle. Modifiable lifestyle risk factors tend to be associated with multiple disease outcomes (for example, physical inactivity is associated with an increased risk of cardiovascular disease, diabetes, and some cancers), and the common risk factors for NCDs tend to cluster within populations. Thus, the combined effects of modifiable risk factors need to be understood before effective public health programs to prevent NCDs can be designed. Here, using data from a large group of middle-aged and elderly Australians (the 45 and Up Study cohort), the researchers develop a lifestyle risk index (score) and examine the association between this score and all-cause mortality (death from any cause). They also identify the most commonly occurring combinations of health risk behaviors and quantify the risk for all-cause mortality for each combination of risk behaviors.

**What Did the Researchers Do and Find?:**

The researchers used mortality registration data to ascertain all-cause mortality during six years of follow-up among 231,048 Australians aged 45 years or older who had completed a lifestyle questionnaire at baseline. They scored six health behaviors reported in the questionnaires (smoking, alcohol use, dietary behavior, physical activity, sedentary behavior, and sleep) for each participant and summed these scores to provide a lifestyle risk index. About a third of the participants reported exposure to no risk factors; about a third, a fifth, and a tenth reported exposure to one, two, and three or more risk factors, respectively. Statistical analysis indicated that exposure to multiple lifestyle risk factors was associated with increased all-cause mortality, that the index score was a good predictor of all-cause mortality, and that the population attributable risk was 31.3%. That is, a third of the person-years lost due to death could have been avoided if all the study participants had had a risk score of zero, provided all six risk factors are causal (responsible for illness and death). More than 90% of the participants had one of the 30 most commonly occurring combinations of risk factors (out of 96 possible combinations). Notably, combinations involving physical inactivity, sedentary behavior, and/or long sleep duration and combinations involving smoking and high alcohol consumption were most strongly associated with all-cause mortality.

**What Do These Findings Mean?:**

Several aspects of the study design (for example, the reliance on self-reported exposure to risk factors and the lack of data on long-term patterns of lifestyle behaviors) may limit the accuracy of these findings. Confounding (people who reported exposure to a specific risk factor may have shared another characteristic that was actually responsible for their illness or death) and reverse causation (a reported behavior may have been caused by an underlying illness, rather than the behavior causing that illness) may also affect the findings’ accuracy. Nevertheless, this study reaffirms the importance of middle-aged and elderly people adopting healthy lifestyles and establishes prolonged sitting and unhealthy sleep duration as two additional risk factors for all-cause mortality that should be included in scores designed to quantify health risk. Finally, the finding that some combinations of health risk behaviors may be more harmful than others suggests that some risk factors have interactive (synergistic) effects on health outcomes. This information may help to guide the design of effective programs for the prevention of NCDs.

**Additional Information:**

This list of resources contains links that can be accessed when viewing the PDF on a device or via the online version of the article at http://dx.doi.org/10.1371/journal.pmed.1001917.

- The US Centers for Disease Control and Prevention (CDC) provides information on all aspects of healthy living, on chronic disease prevention and health promotion, and on noncommunicable diseases around the world; web pages provided by CDC’s Division of Nutrition, Physical Activity, and Obesity include information on staying healthy by exercising and eating a healthy diet, instructional videos, and personal success stories (some information in English and Spanish)
- The World Health Organization provides information about noncommunicable diseases and stories about noncommunicable disease prevention around the world (in several languages); its *Global Recommendations on Physical Activity for Health* is available in several languages; its Global Noncommunicable Disease Network (NCDnet) provides information about the four most common NCDs and about four modifiable risk factors for these diseases; the WHO webpage Face to Face with Chronic Disease is a selection of personal stories from around the world about dealing with NCDs
- More information about the 45 and Up Study is available

## Introduction

Noncommunicable disease is the leading cause of death worldwide [1]. Many noncommunicable diseases, such as cardiovascular disease, some cancers, and diabetes, can be largely attributed to modifiable lifestyle risk factors [2]. Hence, substantial disease, mortality, and economic burden could be prevented through modification of lifestyle behaviors [3–5].

Mounting evidence has implicated lifestyle risk behaviors, such as smoking [6], alcohol use [7], physical inactivity [8], and poor diet [9], in adverse health outcomes. As these common risk behaviors are often associated with multiple disease outcomes and tend to cluster within populations [10], understanding the combined effects of these risk factors on disease burden could be informative for policy making and resource allocation in the context of primary prevention [11].

A number of studies have examined combinations of lifestyle risk factors in relation to disease or mortality outcomes. These studies have mainly focused on the combined effects of smoking, alcohol consumption, physical activity, and diet, and some also included weight status. A recent meta-analysis based on 15 cohort studies found that having a combination of at least four healthy lifestyle factors was associated with a 66% reduction in all-cause mortality [12]. In the past few years, there has been emerging evidence on novel lifestyle risk factors. For example, sedentary behavior (i.e., prolonged sitting, as different from lack of physical activity) was found to be a risk factor for a range of cardiovascular and metabolic diseases and mortality, independent of moderate-to-vigorous-intensity physical activity [13,14]. Recent systematic reviews have also identified short and long sleep duration as predictors of type 2 diabetes [15], cardiovascular disease [16], and all-cause mortality [17]. As such research evidence continues to accumulate, incorporating these new risk factors into lifestyle risk indices will provide more clinically relevant information on combinations of lifestyle risk factors [18].

Using a large Australian cohort, the current study explores a broad range of lifestyle risk behaviors, including habitual sitting time and sleep duration. The objectives of this study are (1) to examine the association between a lifestyle risk index and all-cause mortality, and to quantify the population attributable risk associated with the risk score, and (2) to describe the most commonly occurring combinations of lifestyle risk behaviors, and to quantify the risk for all-cause mortality for each unique lifestyle combination.

## Methods

### Sampling and Procedures

The analyses are based on data from the 45 and Up Study [19], a large-scale (*n* = 267,079) population-based prospective cohort of men and women aged 45 y or older living in the state of New South Wales (NSW), Australia. Baseline data collection was conducted between February 1, 2006, and April 30, 2009. Eligible individuals were randomly sampled from the general population of NSW through the Medicare Australia database and were mailed an invitation to participate, an information leaflet, the study questionnaire, a consent form, and a prepaid reply envelope. Participants joined the study by completing the sex-specific questionnaire and the consent form and mailing them back to the study coordinating center. The final working sample size of the 45 and Up Study corresponded to 11% of the entire NSW population in the target age group. The estimated response rate to the mailed invitations was around 18%, though the exact participation rate could be higher as some individuals may not have received the invitations because of incorrect address or other reasons [19,20]. This study was approved by the NSW Population and Health Services Research Ethics Committee (reference No. 2010/05/234).

### Measures

#### Outcome variable

**Fig 1:**
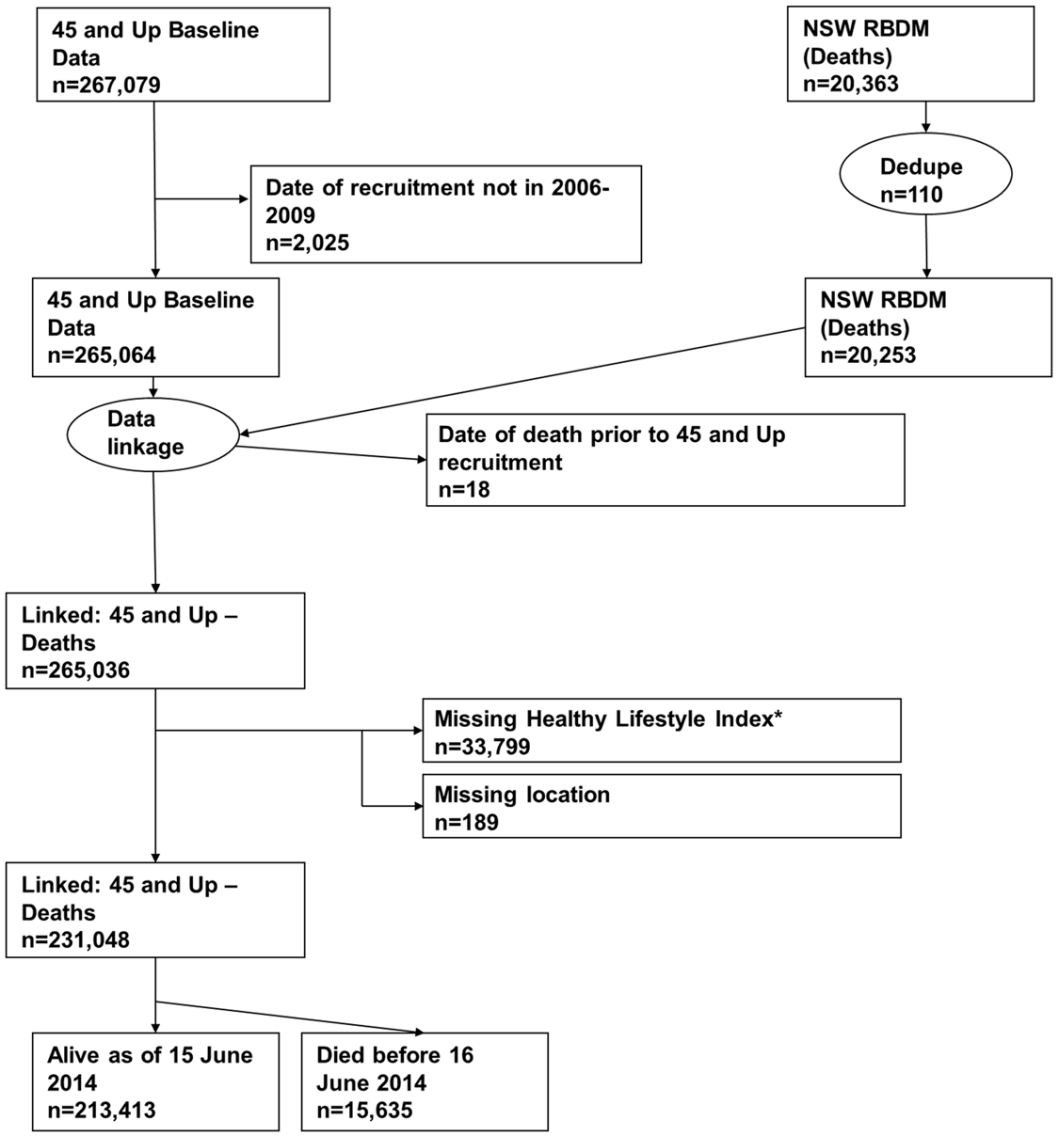
Participant flow diagram. *A risk index including smoking, alcohol use, dietary behavior, physical activity, sedentary behavior, and sleep duration. Dedupe, deduplicated.

All-cause mortality was ascertained from the NSW Registry of Births, Deaths and Marriages (RBDM) from February 1, 2006, to June 15, 2014. The mortality data were linked to the 45 and Up Study baseline data by the Centre for Health Record Linkage (NSW, Australia) using probabilistic record linkage methods and commercially available software (Choice-Maker, ChoiceMaker Technologies). We excluded participants with a missing date of recruitment or a self-reported date of recruitment that was outside the recruitment period. In the RBDM database, we removed any duplicated records of death and retained the earliest record. We then joined the two datasets (45 and Up Study baseline data and RBDM data) and excluded the following participants/records: (1) any records from the RBDM that did not match the 45 and Up Study data, (2) records in which the date of death occurred prior to the date of recruitment into the 45 and Up Study, (3) participants with missing area of residence, and (4) participants with missing lifestyle risk index score. Fig 1 presents the study flow and the sample size at each stage of exclusion. We summarized follow-up time using the median of the reverse Kaplan-Meier estimate of potential follow-up [21].

#### Independent variables

Participants reported on a range of lifestyle risk behaviors in the questionnaire. Smoking status was derived from two questions: “Have you ever been a regular smoker?” and “Are you a regular smoker now?” Participants were asked, “About how many alcoholic drinks do you have each week?” with one drink defined as one glass of wine, one half pint of beer, or one shot of spirits. Dietary behavior was measured by a previously used index [18] of five food items (vegetable, fruit, fish, processed meat, and types of milk) based on the Dietary Guidelines for Australians [22], as an indicator for overall dietary behavior. Physical activity was measured using the Active Australia Survey [23], which has acceptable reliability (Spearman’s rho for test–retest reliability was 0.56–0.64, with 76% agreement on meeting the recommended physical activity level) and validity (Spearman’s rho for total minutes/week of moderate-to-vigorous physical activity was 0.52 against accelerometer measures) [24]. This instrument asked the total time one spent on walking, moderate-intensity, and vigorous-intensity physical activity (bouts of at least 10 min) in the previous week. Sedentary behavior was assessed using a single-item measure: hours spent sitting in a typical 24-h day. This question was adapted from the widely used International Physical Activity Questionnaire [25] and had acceptable reliability and validity [26]. A similar question was also asked about sleep duration in a 24-h day, and this question was comparable with single-item instruments of self-reported sleep duration used by previous studies [27,28]. The specific coding of these lifestyle risk behaviors is presented in Table 1.

**Table 1:** Scoring of risk factors in the lifestyle risk index based on the 45 and Up Study.

Health Behavior	Scoring Method (1 = At Risk, 0 = Not at Risk)	Percentage “At Risk”
Smoking	1 = current smoker	7.2%
Alcohol use	1 = consuming >14 drinks per week (one drink = one glass of wine, one half pint of beer, or one shot of spirits)^a^	19.1%
Dietary behavior	1 = scoring <6 in a dietary index (0–10) consisting of five food items (vegetable, fruit, fish, processed meat, and types of milk)^b^ [18]	17.2%
Physical activity	1 = engaging in <150 min/wk of moderate-to-vigorous-intensity physical activity^c^	22.9%
Sedentary behavior	1 = sitting for >7 h/d^d^	25.0%
Sleep duration	1 = sleeping for <7 or >9 h/d^e^	23.1%

^a^“At risk” corresponds to not adhering to the Australian Government National Health and Medical Research Council recommendation for alcohol consumption [29].

^b^Food items based on the Dietary Guidelines for Australians [22].

^c^“At risk” corresponds to not meeting the WHO recommended level of physical activity [30].

^d^Based on a recent meta-analysis of total sedentary behavior and all-cause mortality [31].

^e^Cutoff points based on a recent meta-analysis on sleep duration and all-cause mortality [17].

Each behavior was coded as 1 (at risk) or 0 (not at risk) and was summed as an index (total score range 0–6). Obesity was not included in the index because it was not considered a behavior, but rather an intermediate health outcome influenced by several of the included lifestyle behaviors.

#### Covariates

The following variables were examined as covariates: age group, sex, educational attainment (school certificate or lower; higher school, trade, or diploma; university degree or higher), marital status (married/cohabitating versus single/divorced/separated/widowed), country of birth (Australia versus other countries), and area of residence based on the Accessibility/Remoteness Index of Australia (major city versus regional/remote) [32].

#### Effect modifiers

Potential effect modifiers included age, sex, educational attainment (as an indicator for socioeconomic status), and body mass index (BMI) (categorized as normal weight/underweight, overweight, obese). In addition, we created a dichotomous variable for cardiovascular or metabolic disease, based on the self-report (at baseline) of (1) physician-diagnosed thrombosis, diabetes, heart disease, or stroke or (2) recent treatment (in the last month) for thrombosis, myocardial infarction, or any other type of heart disease. We created an additional dichotomous variable for recent cancer diagnosis (except for non-melanoma skin cancer), based on self-report for the 10 y prior to baseline data collection.

### Statistical Analysis

All statistical analyses were performed using SAS 9.3 (SAS Institute). Cox proportional hazards analysis was used to examine the association between the lifestyle risk index and all-cause mortality. Adjusted hazard ratios (HRs) and 95% confidence intervals (CIs) were reported. The outcome variable was survival time, which was measured as the time lapse (in weeks) from the date of baseline data collection to death or censoring (June 15, 2014). All Cox proportional hazards regression models were adjusted for sex, age, educational attainment, marital status, country of birth, and area of residence, with covariates classified categorically as per Table 2. Sensitivity analyses were conducted with further adjustment for (1) the presence of cardiovascular or metabolic disease, (2) total number of chronic diseases and/or conditions, and (3) BMI. Participants with a missing value on socio-demographic covariates were included in the analysis using a missing indicator approach. Prior to the analysis, we tested the proportional hazards assumption for the adjustment variables by inspecting plots of cumulative Martingale residuals and the results of a Kolmogorov-type supremum test, based on 1,000 simulations of the residuals [33]. We found no evidence that the proportional hazards assumption was violated (all tests had *p* > 0.30).

**Table 2:** Socio-demographic and health characteristics of adults by lifestyle risk index score in New South Wales, Australia (2006–2009, *n* = 231,048).

Variable	Subcategory	Lifestyle Risk Index Score, *n* (Column Percentage)							Total *n*
		0	1	2	3	4	5	6	
**Sex**	Male	25,771 (35.8)	39,446 (46.5)	27,722 (56.0)	11,895 (63.2)	3,293 (68.0)	616 (74.9)	71 (82.6)	108,814
	Female	46,288 (64.2)	45,443 (53.5)	21,796 (44.0)	6,935 (36.8)	1,551 (32.0)	206 (25.1)	15 (17.4)	122,234
**Age**	45–64 y	45,509 (63.2)	53,484 (63.0)	31,605 (63.8)	12,018 (63.8)	3281 (67.7)	584 (71.1)	58 (67.4)	146,539
	65–79 y	21,715 (30.1)	24,088 (28.4)	12,580 (25.4)	4,462 (23.7)	989 (20.4)	185 (22.5)	21 (24.4)	64,040
	80+ y	4,835 (6.7)	7,317 (8.6)	5,333 (10.8)	2,350 (12.5)	574 (11.9)	53 (6.5)	7 (8.1)	20,469
**Marital status**	Married/cohabitating	57,322 (79.6)	65,390 (77.0)	36,473 (73.7)	12,875 (68.4)	3,092 (63.8)	447 (54.4)	43 (50.0)	175,642
	Single/divorced/separated/windowed	14,443 (20.0)	19,060 (22.5)	12,762 (25.8)	5,838 (31.0)	1,725 (35.6)	369 (44.9)	43 (50.0)	54,240
	Missing	294 (0.4)	439 (0.5)	283 (0.6)	117 (0.6)	27 (0.6)	6 (0.7)	0 (0.0)	1,166
**Educational attainment**	Low (school certificate or lower)	22,256 (30.9)	26,582 (31.3)	16,216 (32.8)	6,837 (36.3)	2,013 (41.6)	386 (47.0)	46 (53.5)	74,336
	Middle (higher school/trade/diploma)	30,880 (42.9)	35,803 (42.2)	20,911 (42.2)	7,943 (42.2)	1,994 (41.2)	324 (39.4)	29 (33.7)	97,884
	High (university degree or higher)	18,151 (25.2)	21,446 (25.3)	11,710 (23.7)	3,768 (20.0)	748 (15.4)	92 (11.2)	9 (10.5)	55,924
	Missing	772 (1.1)	1,058 (1.3)	681 (1.4)	282 (1.5)	89 (1.8)	20 (2.4)	2 (2.3)	2,904
**Area of residence**	Major city	30,832 (42.8)	38,228 (45.0)	22,865 (46.2)	8,717 (46.3)	2,129 (44.0)	322 (39.2)	23 (26.7)	103,116
	Regional/remote	41,227 (57.2)	46,661 (55.0)	26,653 (53.8)	10,113 (53.7)	2,715 (56.1)	500 (60.8)	63 (73.3)	127,932
**Country of birth**	Australia	54,518 (75.7)	63,838 (75.2)	36,901 (74.5)	14,262 (75.7)	3,778 (78.0)	653 (79.4)	67 (77.9)	174,017
	Other country	17,541 (24.3)	21,051 (24.8)	12,617 (25.5)	4,568 (24.3)	1,066 (22.0)	169 (20.6)	19 (22.1)	57,031
**Cardiovascular or metabolic disease** ^a^	Yes	15,583 (21.6)	19,957 (23.5)	12,765 (25.8)	5,272 (28.0)	1,399 (28.9)	229 (27.9)	30 (34.9)	55,235
	No	56,476 (78.4)	64,932 (76.5)	36,753 (74.2)	13,558 (72.0)	3,445 (71.1)	593 (72.1)	56 (65.1)	175,813
**Cancer diagnosis in past 10 y** ^b^	Yes	5,839 (8.1)	6,868 (8.1)	4,140 (8.4)	1,654 (8.8)	401 (8.3)	82 (10.0)	7 (8.1)	18,991
	No	66,220 (91.9)	78,021 (91.9)	45,378 (91.6)	17,176 (91.2)	4,443 (91.7)	740 (90.0)	79 (91.9)	212,057
**Total *n***		72,059	84,889	49,518	18,830	4,844	822	86	231,048

^a^Self-reported physician-diagnosed thrombosis, diabetes, heart disease, or stroke or recent treatment (in the last month) for thrombosis, myocardial infarction, or any other type of heart disease.

^b^Self-reported cancer diagnosis (except for non-melanoma skin cancer) within the 10 y prior to baseline data collection.

First, we tested independent associations between each individual risk behavior and survival time adjusted for other lifestyle risk behaviors and socio-demographic covariates, based on the log-likelihood test of including these in the model. Then, we tested the lifestyle risk index as the exposure variable, adjusted for all covariates, and presented the *c* index for risk discrimination/prediction. The *c* index is defined as the proportion of all possible pairs of participants whose predictions and outcomes are concordant. Therefore, the *c* index can be interpreted as the probability that the predicted risk is higher in those who die sooner; the *c* index score ranges from 0.5 (no discrimination) to 1.0 (perfect discrimination) [34].

Based on the model with the lifestyle risk index as the exposure variable, we tested potential effect modification and presented stratified analyses by age group, sex, educational attainment, BMI, whether individuals were diagnosed with cardiovascular or metabolic disease, and whether individuals were diagnosed with cancer within the past 10 y. Finally, we calculated the partial population attributable risk (PAR_p_) for the lifestyle risk index score, which can be interpreted as the proportion of survival time that could have been added if all participants had a risk index score of zero, adjusted for all covariates [35]. To account for “reverse causality” due to occult disease at baseline, the main statistical analysis was repeated excluding deaths that occurred within the first 2 y of follow-up.

Finally, to examine specific patterns of lifestyle risk behaviors, 96 variables representing all possible mutually exclusive combinations of smoking, high alcohol intake, physical inactivity, poor diet, prolonged sitting, and short/long sleep duration were created. Short and long sleep durations were separated as two different risk factors, as their associations with mortality may be explained by different mechanisms [17]. We present the prevalence and HR (95% CI) for each combination; we repeated the analysis excluding deaths within the first 2 y as an additional sensitivity analysis.

## Results

### Descriptive Statistics

We linked 20,253 records of death from the RBDM to 265,064 participant records from the 45 and Up Study (Fig 1). The final sample for analyses included 231,048 participants, of whom 15,635 died prior to June 15, 2014. S1 Table compares the characteristics and the mortality outcomes of the final analytical sample with those of individuals excluded because of missing lifestyle risk index score. Compared with the analytical sample, those who were excluded were older at baseline and more likely to die during the follow-up. The cohort had a median potential follow-up time of 5.9 y (mean recorded follow-up time 6.1 y), with a total of 1,409,591 person-years of follow-up before death or censoring. Overall, at baseline, 36.6% of the participants were aged 65 y or older, 52.9% were female, 76.0% were married/cohabitating, 24.2% had a university degree, 44.6% lived in a major city, and 75.3% were born in Australia (Table 2).

At baseline, 7.2% of study participants were smokers, 19.1% consumed more than 14 drinks of alcohol per week, 22.9% were not meeting physically activity recommendations, 17.2% were classified as having poor diet, 25.0% sat for more than 7 h per day, and 23.1% slept too little or too much (Table 1). Overall, 31.2% of participants reported no risk behavior (lifestyle risk index score = 0), 36.7% had one risk behavior, and 21.4%, 8.1%, 2.1%, 0.4%, and 0.04% had a lifestyle risk index score of 2, 3, 4, 5, and 6, respectively. Higher lifestyle risk index scores were more prevalent among males, those aged 80+ y, those who were not married/cohabitating, and those with lower educational attainment.

### Individual Risk Behavior and All-Cause Mortality

When all six dichotomized individual risk behaviors were entered in the model with all covariates, five showed independent associations with all-cause mortality. Of them, smoking (HR = 1.94, 95% CI 1.82–2.06) and physical inactivity (HR = 1.72, 95% CI 1.66–1.77) had the strongest association with mortality, followed by prolonged sitting (HR = 1.33, 95% CI 1.29–1.38), short/long sleep (HR = 1.31, 95% CI 1.27–1.36), and poor diet (HR = 1.11, 95% CI 1.07–1.15). There was no significant association between high alcohol intake and all-cause mortality (HR = 0.98, 95% CI 0.94–1.02).

### Lifestyle Risk Index and All-Cause Mortality

The results from the Cox proportional hazards regression analyses show the association between the lifestyle risk index score and all-cause mortality adjusted for age, sex, educational attainment, marital status, country of birth, and area of residence (Table 3). All-cause mortality HRs compared to individuals without lifestyle risk factors were 1.27 for those with one risk factor, and 1.73, 2.45, 3.06, 4.61, and 5.38 for those with 2, 3, 4, 5, and 6 risk factors, respectively. There was a positive relationship between lifestyle risk index score and all-cause mortality, though the HRs for those with a score of 5 or 6 had wide confidence intervals because of small sample sizes. Additional sensitivity analyses adjusted for BMI, physician-diagnosed cardiovascular and metabolic disease, diagnosis of cancer in the past 10 y, and the total number of chronic diseases/conditions yielded minimal (<2%) change in HRs. Overall, the lifestyle risk index showed good prediction of all-cause mortality (*c* index = 0.763, 95% CI 0.749–0.776). The PAR_p_ calculated based on the overall study sample was 31.3% (95% CI 27.6%–34.9%), which indicates that if other variables were held constant, 31.3% of survival time could have been added if all participants had a risk index score of zero.

**Table 3:** Crude cumulative death rates and adjusted hazard ratios for all-cause mortality by lifestyle risk index score among a population-based Australian sample of adults from the 45 and Up Study (2006–2014, *n* = 231,048).

Sample	Lifestyle Risk Index Score						
	0	1	2	3	4	5	6
**All participants (*n* = 231,048)**							
Cumulative death rate	4.15%	5.90%	8.75%	12.87%	14.74%	17.40%	23.26%
HR (95% CI)	Reference	1.27 (1.21–1.33)	1.73 (1.65–1.81)	2.45 (2.32–2.59)	3.06 (2.82–3.33)	4.61 (3.90–5.46)	5.38 (3.46–8.35)
**Participants with more than 2 y of follow–up (*n* = 227,346)**							
Cumulative death rate	3.34%	4.76%	6.71%	9.40%	10.57%	12.50%	18.52%
HR (95% CI)	Reference	1.26 (1.20–1.33)	1.64 (1.56–1.73)	2.23 (2.09–2.37)	2.76 (2.50–3.05)	4.18 (3.41–5.13)	5.44 (3.27–9.04)
**Aged 45–64 y (*n* = 146,539)**							
Cumulative death rate	1.26%	1.65%	2.28%	3.63%	5.15%	9.08%	8.62%
HR (95% CI)	Reference	1.20 (1.08–1.33)	1.52 (1.36–1.70)	2.17 (1.91–2.46)	2.80 (2.35–4.18)	4.26 (3.20–5.66)	3.59 (1.48–8.66)
**Aged 65–79 y (*n* = 64,040)**							
Cumulative death rate	5.95%	8.27%	11.89%	17.93%	22.04%	30.81%	47.62%
HR (95% CI)	Reference	1.32 (1.23–1.42)	1.82 (1.68–1.96)	2.66 (2.43–2.91)	3.25 (2.81–3.75)	4.75 (3.64–6.20)	6.61 (3.54–12.33)
**Aged 80+ y (*n* = 20,469)**							
Cumulative death rate	23.27%	29.18%	39.66%	50.55%	56.97%	62.26%	71.43%
HR (95% CI)	Reference	1.25 (1.17–1.35)	1.80 (1.67–1.93)	2.53 (2.33–2.75)	3.01 (2.66–3.41)	3.27 (2.31–4.63)	4.10 (1.70–9.87)
**Men (*n* = 108,814)**							
Cumulative death rate	6.71%	7.80%	10.13%	13.25%	14.94%	18.34%	21.13%
HR (95% CI)	Reference	1.20 (1.13–1.28)	1.61 (1.52–1.71)	2.12 (1.98–2.27)	2.63 (2.37–2.91)	4.07 (3.36–4.93)	4.52 (2.72–7.53)
**Women (*n* = 122,234)**							
Cumulative death rate	2.73%	4.25%	6.99%	12.23%	14.31%	14.56%	33.33%
HR (95% CI)	Reference	1.36 (1.26–1.46)	1.92 (1.78–2.07)	3.18 (2.91–3.48)	4.13 (3.57–4.77)	6.30 (4.38–9.06)	8.78 (3.64–21.15)
**Education: low (school certificate or lower) (*n* = 74,336)**							
Cumulative death rate	5.00%	7.55%	11.57%	17.24%	19.13%	18.13%	21.74%
HR (95% CI)	Reference	1.30 (1.21–1.40)	1.81 (1.68–1.95)	2.75 (2.53–2.99)	3.37 (2.99–3.79)	4.90 (3.84–6.25)	5.48 (2.94–10.22)
**Education: middle (higher school/trade/diploma) (*n* = 97,884)**							
Cumulative death rate	4.25%	5.87%	8.14%	11.34%	11.89%	17.59%	20.69%
HR (95% CI)	Reference	1.28 (1.19–1.37)	1.70 (1.58–1.83)	2.31 (2.12–2.52)	2.95 (2.57–3.39)	4.46 (3.42–5.83)	3.58 (1.60–7.99)
**Education: high (university degree or higher) (*n* = 55,924)**							
Cumulative death rate	2.73%	3.63%	5.29%	7.14%	9.09%	9.78%	33.33%
HR (95% CI)	Reference	1.21 (1.08–1.35)	1.65 (1.47–1.86)	2.14 (1.84–3.07)	2.37 (1.84–3.07)	3.42 (1.77–6.62)	16.34 (5.23–51.01)
**Normal weight/underweight (*n* = 81,728)**							
Cumulative death rate	4.50%	6.85%	11.27%	17.63%	20.07%	23.03%	28.57%
HR (95% CI)	Reference	1.31 (1.22–1.41)	1.91 (1.78–2.05)	2.90 (2.67–3.16)	3.56 (3.14–4.04)	6.02 (4.75–7.63)	4.90 (2.63–9.15)
**Overweight (*n* = 85,747)**							
Cumulative death rate	3.99%	5.23%	7.39%	10.16%	12.69%	13.58%	16.67%
HR (95% CI)	Reference	1.31 (1.22–1.41)	1.55 (1.43–1.68)	2.07 (1.88–2.29)	2.97 (2.56–3.45)	3.40 (2.40–4.82)	5.05 (1.89–13.48)
**Obese (*n* = 47,822)**							
Cumulative death rate	3.48%	4.95%	6.84%	9.84%	9.47%	13.27%	22.73%
HR (95% CI)	Reference	1.29 (1.15–1.45)	1.71 (1.52–1.92)	2.29 (2.01–2.62)	2.31 (1.89–2.82)	4.11 (2.76–6.12)	7.48 (3.09–18.09)
**Participants with cardiovascular or metabolic disease** ^a^ **(*n* = 55,235)**							
Cumulative death rate	8.89%	12.43%	18.03%	25.06%	26.30%	31.00%	33.33%
HR (95% CI)	Reference	1.28 (1.20–1.37)	1.78 (1.66–1.90)	2.42 (2.24–2.62)	2.93 (2.61–3.29)	4.56 (3.59–5.79)	6.01 (3.22–11.21)
**Participants without cardiovascular or metabolic disease (*n* = 175,813)**							
Cumulative death rate	2.84%	3.89%	5.53%	8.14%	10.04%	12.14%	17.86%
HR (95% CI)	Reference	1.24 (1.16–1.32)	1.63 (1.53–1.75)	2.38 (2.20–2.57)	3.07 (2.73–3.46)	4.48 (3.54–5.68)	4.70 (2.52–8.76)
**Participants with cancer diagnosis in past 10 y** ^b^ **(*n* = 18,991)**							
Cumulative death rate	10.52%	13.82%	18.96%	25.51%	28.93%	30.49%	14.29%
HR (95% CI)	Reference	1.18 (1.07–1.31)	1.55 (1.39–1.72)	1.99 (1.76–2.26)	2.46 (2.01–3.01)	3.38 (2.26–5.06)	1.83 (0.26–13.00)
**Participants without cancer diagnosis in past 10 y (*n* = 212,057)**							
Cumulative death rate	3.59%	5.21%	7.82%	11.66%	13.46%	15.95%	24.05%
HR (95% CI)	Reference	1.29 (1.22–1.35)	1.77 (1.68–1.86)	2.54 (2.39–2.70)	3.19 (2.91–3.49)	4.83 (4.01–5.82)	6.10 (3.88–9.58)

HRs adjusted for age, sex, educational attainment, marital status, area of residence, and country of birth.

^a^Self-reported physician-diagnosed thrombosis, diabetes, heart disease, or stroke or recent treatment (in the last month) for thrombosis, myocardial infarction, or any other type of heart disease.

^b^Self-reported cancer diagnosis (except for non-melanoma skin cancer) within the 10 y prior to baseline data collection.

Analysis of effect modification showed that the association between lifestyle risk index score and all-cause mortality did not vary significantly by age category and did not differ among those with and without cardiovascular or metabolic disease. However, there were significant interactions between lifestyle risk index score and sex (χ^2^ = 65.9, *p* < 0.0001), educational attainment (χ^2^ = 42.4, *p* = 0.011), BMI (χ^2^ = 24.5, *p* < 0.001), and cancer diagnosis in the past 10 y (χ^2^ = 31.8, *p* < 0.0001). Stratified analysis suggested a stronger association between lifestyle risk index score and all-cause mortality among women, those with lower educational attainment, those with normal weight/underweight, and those without a cancer diagnosis in the past 10 y (Table 3). Finally, when the analysis was repeated among those with more than 2 y of follow-up, the magnitude of the association between lifestyle risk index score and all-cause mortality was similar, but slightly attenuated.

### Combinations of Risk Behaviors

Table 4 presents all 96 mutually exclusive combinations of risk behaviors. Of these, the 30 most commonly occurring combinations accounted for more than 90% of all participants. Compared to being without any risk behavior, the majority of risk combinations were associated with a significantly elevated risk for all-cause mortality. Among those with one risk factor, the most common single risk behavior was prolonged sitting time (9.1%), followed by physical inactivity (7.1%), unhealthy diet (6.9%), short sleep duration (5.7%), high alcohol intake (4.2%), and long sleep duration only (2.4%). Among those with at least two risk factors, the most common combinations were physical inactivity plus prolonged sitting time (2.9%), followed by poor diet plus prolonged sitting time (2.2%). Out of all the single risk behaviors, smoking had the strongest association with all-cause mortality (HR = 1.90). Among the commonly occurring risk combinations, several showed a relatively strong association with all-cause mortality, such as physical inactivity plus prolonged sitting time (HR = 2.42), physical inactivity plus long sleep duration (HR = 2.68), high alcohol intake plus physical inactivity plus prolonged sitting time (HR = 2.51), physical inactivity plus prolonged sitting time plus short sleep duration (HR = 2.59), physical inactivity plus prolonged sitting time plus long sleep duration (HR = 4.23), smoking plus high alcohol intake (HR = 2.80), and smoking plus high alcohol intake plus short sleep duration (HR = 4.68). Sensitivity analysis excluding deaths within the first 2 y showed similar prevalence and HRs for all risk combinations (S2 Table).

**Table 4:** Prevalence of all 96 combinations of lifestyle risk behaviors and adjusted hazard ratios for their associations with all-cause mortality among middle-aged and older adults based on the 45 and Up Study, Australia (2006–2014, *n* = 231,048).

Lifestyle Risk Behavior							Percent	HR^a^
Smoking	Poor Diet	High Alcohol Intake	Physical Inactivity	Prolonged Sitting	Long Sleep Duration	Short Sleep Duration		
0	0	0	0	0	0	0	31.19	Reference
0	0	0	0	1	0	0	9.13	1.15 (1.07–1.25)
0	0	0	1	0	0	0	7.14	1.61 (1.52–1.72)
0	1	0	0	0	0	0	6.89	1.04 (0.96–1.13)
0	0	0	0	0	0	1	5.66	1.09 (1.00–1.18)
0	0	1	0	0	0	0	4.18	1.08 (0.99–1.19)
0	0	0	1	1	0	0	2.92	2.42 (2.24–2.61)
0	0	0	0	0	1	0	2.35	1.44 (1.31–1.57)
0	1	0	0	1	0	0	2.24	1.08 (0.94–1.25)
0	0	0	0	1	0	1	1.75	1.19 (1.02–1.38)
0	0	0	1	0	0	1	1.75	1.60 (1.45–1.78)
0	0	1	1	0	0	0	1.60	1.80 (1.62–2.00)
0	1	1	0	0	0	0	1.55	1.21 (1.06–1.40)
0	0	1	0	1	0	0	1.47	1.35 (1.16–1.58)
1	0	0	0	0	0	0	1.39	1.90 (1.61–2.25)
0	1	0	1	0	0	0	1.15	1.56 (1.37–1.78)
0	0	0	1	0	1	0	1.11	2.68 (2.45–2.92)
0	0	1	0	0	0	1	0.92	1.25 (1.05–1.49)
0	1	0	0	0	0	1	0.92	1.01 (0.84–1.23)
0	0	1	1	1	0	0	0.75	2.51 (2.19–2.86)
0	0	0	1	1	0	1	0.73	2.59 (2.26–2.96)
0	1	0	0	0	1	0	0.68	1.44 (1.24–1.68)
1	0	1	0	0	0	0	0.63	2.80 (2.30–3.40)
0	0	0	1	1	1	0	0.57	4.23 (3.86–4.64)
0	1	1	0	1	0	0	0.54	1.06 (0.80–1.40)
0	1	0	1	1	0	0	0.52	2.16 (1.81–2.58)
1	1	0	0	0	0	0	0.51	2.00 (1.54–2.60)
0	0	0	0	1	1	0	0.51	1.95 (1.68–2.26)
0	0	1	0	0	1	0	0.43	1.54 (1.29–1.83)
0	0	1	1	0	0	1	0.42	1.99 (1.67–2.39)
1	0	0	0	0	0	1	0.41	2.65 (2.02–3.49)
0	1	1	1	0	0	0	0.40	1.64 (1.31–2.05)
1	0	0	1	0	0	0	0.40	2.15 (1.67–2.78)
1	0	0	0	1	0	0	0.38	2.42 (1.80–3.26)
0	1	0	0	1	0	1	0.37	0.99 (0.69–1.41)
0	0	1	0	1	0	1	0.35	1.59 (1.19–2.13)
1	1	1	0	0	0	0	0.35	1.86 (1.38–2.51)
0	0	1	1	0	1	0	0.32	2.95 (2.57–3.38)
1	0	1	1	0	0	0	0.25	2.61 (1.92–3.56)
0	1	1	0	0	0	1	0.25	1.30 (0.94–1.80)
1	0	1	0	0	0	1	0.22	4.68 (3.48–6.28)
0	1	1	0	0	1	0	0.22	1.47 (1.13–1.91)
0	1	0	1	0	0	1	0.21	1.49 (1.11–2.00)
0	1	0	1	0	1	0	0.21	2.28 (1.87–2.78)
0	0	1	1	1	0	1	0.21	2.39 (1.88–3.04)
0	1	1	1	1	0	0	0.20	2.07 (1.53–2.78)
1	0	1	0	1	0	0	0.19	3.19 (2.26–4.50)
0	0	1	1	1	1	0	0.18	4.23 (3.63–4.91)
1	0	0	1	1	0	0	0.17	2.93 (2.12–4.05)
1	1	0	0	1	0	0	0.15	2.70 (1.79–4.07)
0	1	0	0	1	1	0	0.14	1.94 (1.47–2.56)
1	0	1	1	1	0	0	0.14	4.76 (3.48–6.50)
1	0	0	1	0	0	1	0.13	3.90 (2.72–5.60)
1	1	0	1	0	0	0	0.12	2.73 (1.81–4.12)
1	0	0	0	1	0	1	0.11	3.93 (2.53–6.10)
1	0	0	0	0	1	0	0.11	2.06 (1.37–3.11)
0	0	1	0	1	1	0	0.11	1.82 (1.35–2.47)
0	1	0	1	1	0	1	0.10	3.72 (2.68–5.17)
1	1	1	0	1	0	0	0.10	2.93 (1.82–4.72)
1	0	1	1	0	0	1	0.10	3.22 (2.03–5.13)
0	1	1	1	0	0	1	0.10	2.01 (1.31–3.08)
1	1	1	1	0	0	0	0.10	1.77 (1.01–3.13)
0	1	1	0	1	0	1	0.10	1.55 (0.86–2.81)
1	1	0	0	0	0	1	0.10	3.40 (2.08–5.56)
0	1	1	1	0	1	0	0.09	2.02 (1.48–2.75)
1	0	1	0	1	0	1	0.09	3.69 (2.18–6.24)
1	1	1	0	0	0	1	0.08	2.85 (1.65–4.91)
0	1	0	1	1	1	0	0.08	3.33 (2.59–4.28)
1	0	1	0	0	1	0	0.08	3.48 (2.26–5.35)
1	0	0	1	1	0	1	0.06	4.24 (2.67–6.74)
1	0	0	1	0	1	0	0.06	4.86 (3.43–6.88)
1	1	0	1	1	0	0	0.06	2.86 (1.53–5.31)
1	0	1	1	0	1	0	0.06	3.55 (2.20–5.72)
1	0	1	1	1	0	1	0.06	4.77 (2.92–7.81)
1	1	1	0	0	1	0	0.05	3.12 (1.77–5.49)
1	1	1	1	1	0	0	0.05	6.40 (4.24–9.65)
1	1	0	0	0	1	0	0.05	3.56 (2.18–5.82)
0	1	1	1	1	0	1	0.05	2.55 (1.51–4.31)
0	1	1	0	1	1	0	0.04	2.04 (1.30–3.21)
0	1	1	1	1	1	0	0.04	2.81 (1.84–4.27)
1	1	0	0	1	0	1	0.04	2.53 (1.05–6.09)
1	0	0	1	1	1	0	0.04	7.61 (5.21–11.12)
1	0	1	1	1	1	0	0.03	9.09 (6.30–13.12)
1	0	0	0	1	1	0	0.03	3.15 (1.50–6.60)
1	1	1	1	0	1	0	0.03	3.64 (1.82–7.30)
1	1	1	0	1	0	1	0.03	6.32 (3.28–12.16)
1	1	0	1	0	0	1	0.03	3.22 (1.34–7.73)
1	1	1	1	0	0	1	0.03	3.60 (1.61–8.02)
1	1	0	1	0	1	0	0.03	3.40 (1.77–6.54)
1	1	1	1	1	0	1	0.02	3.86 (1.93–7.73)
1	0	1	0	1	1	0	0.02	4.29 (2.14–8.59)
1	1	1	1	1	1	0	0.02	7.07 (4.01–12.48)
1	1	0	1	1	0	1	0.02	6.77 (2.54–18.05)
1	1	1	0	1	1	0	0.01	3.40 (1.41–8.17)
1	1	0	0	1	1	0	0.01	2.56 (0.96–6.82)
1	1	0	1	1	1	0	0.01	10.29 (4.90–21.61)

A “1” denotes the presence of the risk behavior, and a “0” denotes the absence of the risk behavior.

^a^Adjusted for age, sex, educational attainment, marital status, area of residence, and country of birth.

## Discussion

In this study, we found that multiple lifestyle risk factors among middle-aged and older Australian adults were associated with an increased risk for all-cause mortality over 6 y of follow-up. There was a clear association between the number of risk factors, as indicated by the lifestyle risk score, and all-cause mortality. Overall, all six risk factors accounted for a third of the person-year loss due to mortality when socioeconomic characteristics were held constant.

Evidence is accumulating on the health effects of the combined behavioral risk factors of smoking, high alcohol intake, poor diet, and physical inactivity. The associations found in the current study are similar to those from previous studies, which tended to have smaller sample sizes and a more limited range of lifestyle risk factors. For example, Ford and colleagues found a strong association between the number of lifestyle risk behaviors (smoking, non-moderate alcohol consumption, smoking, and poor diet based on the Healthy Eating Index) and all-cause mortality in a US-based sample [36]. Khaw et al. found a clear inverse relationship between adherence to four health behaviors (not smoking, being physically active, moderate alcohol intake, and fruit and vegetable intake indicated by plasma vitamin C level) and all-cause mortality in a UK-based sample [37]. These findings have been replicated by a number of epidemiological studies that assessed similar risk factors using various measures [38–43]. Despite the heterogeneous measures, risk classification, sample characteristics, and follow-up time of these studies, the additive nature of the association between risk indices and mortality has been consistent, suggesting the generalizability of these findings. Such evidence is furthered here by adding the new risk factors of prolonged sitting and unhealthy sleep duration.

The validity of our findings was also enhanced through comprehensive sensitivity analyses, where we conducted subgroup analyses, excluded deaths within the first 2 y, and further adjusted for chronic disease and BMI as additional covariates. Despite statistically significant effect modification by sex, educational attainment, BMI, and cancer diagnosis in the past 10 y, the overall difference in effect sizes across subgroups or when adjusting for additional covariates was small, and the patterns of associations were consistent. This reinforces an important message for public health and clinical practice that adherence to low-risk lifestyles is likely to be protective for all.

It is important to acknowledge that not all risk behaviors contribute to mortality similarly and that their combined effects may not be additive. We therefore supplemented risk index analysis with risk combination analysis. This allowed in-depth exploration of interactions among behaviors in relation to all-cause mortality. One compelling observation was that some risk behaviors tend to cluster, particularly in certain patterns, and that the joint risk could be much higher than the sum of the individual risks. For example, smoking was the least common single risk factor (only 1.39% of participants reported only smoking risk), and it was more than four times more likely to occur with other risk factors than on its own. Smoking was also the most “deadly” single risk factor (HR = 1.90). The risk behavior that co-occurred with smoking the most was high alcohol consumption. Though high alcohol intake on its own was not significantly associated with higher mortality risk (HR = 1.08), it augmented the risk noticeably when paired with smoking (HR = 2.80). Furthermore, when these two risk factors co-occurred with short sleep duration, which was marginally associated with all-cause mortality on its own (HR = 1.09), the combined risk was increased dramatically (HR = 4.68). These findings suggest that there is a “synergistic effect” among risk factors and that future epidemiological research and behavioral interventions should take into account the patterns of risk factor co-occurrence and their interactive effects on health outcomes.

A unique contribution of the current study is the inclusion of prolonged sitting and short/long sleep duration as additional risk indicators, which were not reported in previous cohort studies [12]. Growing research evidence on the health effects of sedentary behavior and sleep [13,16,44,45] suggests that both may be important behaviors that together constitute a large proportion of one’s daily life and contribute to chronic disease risk. However, few studies have examined the interactions between these behaviors and other lifestyle risk factors in relation to health outcomes. A key finding that emerged from the current study is that prolonged sitting time alone, as the most common single risk factor, had a small effect on all-cause mortality (HR = 1.15). However, the combination of prolonged sitting time and physical inactivity had a much stronger association with mortality (HR = 2.42). This might indicate that prolonged sitting tends to be particularly harmful among those who are physically inactive. Such interactive effects were noted in a recent meta-analysis, which found that the association between sedentary behavior and health outcomes was more pronounced among those with lower physical activity [46]. When sleep was present as a lone risk factor, short sleep duration was only marginally associated with mortality (HR = 1.09), while long sleep duration was associated with much higher risk (HR = 1.44). Such a pattern of associations was noted in recent meta-analyses [17,47]; one meta-analysis also found that the effect of long sleep duration was stronger in older than younger cohorts [17]. It is biologically plausible that short sleep duration may increase mortality risk through adverse endocrinologic, immunologic, and metabolic effects [48,49,50] or through chronic inflammation [47,51,52]. The mechanism for the association between long sleep duration and mortality is not well understood [17,47]. Most studies suggest that long sleep duration tends to be associated with sleep fragmentation, fatigue, depression, and underlying disease and poor health [53]. Therefore, the observed association between long sleep duration and all-cause mortality could be due to “reverse causality” or residual confounding [17,54]. An interesting observation from the current study is that risk combinations involving long sleep duration, prolonged sitting, and/or physical inactivity tended to be among those with the strongest associations with mortality, with HRs ranging from 2 to above 4. These associations remained significant and of similar magnitude after excluding deaths within the first 2 y of follow-up (S2 Table). This may suggest that the underlying characteristics associated with such behavioral patterns involving long sleep, sedentariness, and inactivity, perhaps not limited to major occult disease or failing health, may have contributed to the elevated risk for morality.

### Strengths and Limitations

The current study is the first to our knowledge to test a lifestyle risk index and multiple behavioral risk combinations incorporating sedentary and sleep behaviors as additional risk factors for all-cause mortality. In rigorous sensitivity analyses, subgroup analyses, and tests of effect modification, the association between the lifestyle risk index and all-cause mortality remained robust, implying internal validity for our findings. Using a large population-based sample allowed us to test all lifestyle risk combinations, which provided unique insights into understanding the complex interacting relationships among lifestyle risk factors, particularly for sedentary behavior and sleep.

However, despite the novelty and methodologic rigor, the findings from this study should be interpreted in the light of the study’s limitations. First, all lifestyle risk behaviors were self-reported, although using established and validated questions. Given that misclassification due to self-report is potentially non-random (i.e., if people tended to report desirable behaviors because of social desirability bias), the results are most likely biased toward the null [36]. Therefore, the potential risk reduction related to the six lifestyle behaviors, as indicated by PAR_p_, is likely to be underestimated. Second, the measures of several risk behaviors are under-specified; for example, the alcohol measure did not capture short-term alcohol risk, such as binge drinking, and could not distinguish non-drinkers from ex-drinkers who might have quit drinking due to prior alcohol-related problems. The dietary measure was limited to a small number of food items. The sleep measure was limited to quantity only, without taking into account other aspects of sleep hygiene or sleep quality. The smoking measure did not take into account past smoking. However, a recent study from the same cohort found a much lower risk for all-cause mortality among past smokers than current smokers, and the mortality risk among those who quit before 45 y of age did not differ significantly from that of never smokers [55]. Furthermore, when we adjusted for past smoking in the main analysis, the results did not change substantially. On the other hand, a strength of this analysis is that it focused on six lifestyle behaviors and did not conflate behaviors with their outcomes, as some lifestyle risk indices have done before by including weight status or other metabolic health indicators in the index [11,56]. Overall, despite limitations in measurement, the use of indices such as ours supports policy-relevant public health recommendations by using categorical thresholds for risk and allowing lifestyle risk to be easily captured and assessed across settings. The third limitation of our study is that it focused on participants’ reports of lifestyle risk behaviors at one time point; therefore, we could not determine the habitual or changing patterns of participants’ behaviors over time. For example, the risk classification for smoking was based on current smoking status only, equating past and never smokers, which could lead to underestimation of the health risk associated with smoking. The same applies to other risk behaviors. Therefore, the current analyses could be further improved by incorporating past behavioral patterns and future waves of follow-up data. Fourth, this study could be further strengthened by including cause-specific mortality outcomes, such as cardiovascular disease mortality, but these data are not yet available for the time period studied. Fifth, although the cohort sample was not representative of the population (participants in the 45 and Up Study were healthier than the general population because of selection bias), a recent study comparing the current cohort with a population representative sample in NSW found similar estimates for the associations between risk factors and health outcomes, despite the difference in the prevalence of risk factors [57]. Furthermore, most prior epidemiological studies have found little evidence for considerable bias attributable to nonparticipation [20]. This reinforces the epidemiological axiom that associations, compared to prevalence, are less dependent on the representativeness of the sample.

### Conclusion

This large study reaffirms the importance of healthy lifestyles, here evidenced for adults aged 45 y and older. This analysis investigated four established and two novel risk factors, namely, prolonged sitting and unhealthy sleep duration, which may be added to behavioral indices or risk combinations to quantify health risk. The prevalent combinations of risk factors suggest new strategic targeting for chronic disease prevention.

## Supporting Information

S1 Table Comparison of participants with a lifestyle risk index score and included in the analysis versus those with a missing score (New South Wales, Australia, *n* = 264,847). (DOC)

S2 Table Sensitivity analysis: prevalence of all 96 combinations of lifestyle risk behaviors and adjusted hazard ratios for their associations with all-cause mortality after excluding deaths within the first 2 y (2006–2014, *n* = 227,346). (DOC)

S1 Text STROBE statement. (DOCX)

S2 Text Statistical analysis plan. (DOC)

## Abbreviations

BMI: body mass index
CI: confidence interval
HR: hazard ratio
NSW: New South Wales
PAR_p_: partial population attributable risk
RBDM: Registry of Births, Deaths and Marriages
