# Supplementary material for: Traditional and Emerging Lifestyle Risk Behaviors and All-Cause Mortality in Middle-Aged and Older Adults: Evidence from a Large Population-Based Australian Cohort
Source: PLoS Med. 2015 Dec 8;12(12):e1001917. doi: 10.1371/journal.pmed.1001917 (PMC4672919; doi:10.1371/journal.pmed.1001917)
Supplement: S1 Table — (DOC) [file pmed.1001917.s001.doc]

| **S1 Table. Comparison of participants with a lifestyle index score included in the analysis versus those with a missing score (New South Wales, Australia, n=264,847)** | | | |
| --- | --- | --- | --- |
| **Variable** | **Value** | **Complete score (n=**231,048) | **Missing score**  **(n=**33,799) |
|  |  | **n (column percentage)** | |
|  |  |  |  |
| Sex | Male | 108,814 (47.1%) | 14,054 (41.6%) |
|  | Female | 122,234 (52.9%) | 19,745 (58.4%) |
|  |  |  |  |
| Age | 45-64 yrs | 146,539 (63.4%) | 15,783 (46.7%) |
|  | 65-79 yrs | 64,040 (27.7%) | 11,521 (34.1%) |
|  | 80+ yrs | 20,469 (8.9%) | 6,495 (19.2%) |
|  |  |  |  |
| Marital status | Married/cohabitating | 175,642 (76.4%) | 22,281 (66.8%) |
|  | Single/divorced/separated/widowed | 54,240 (23.6%) | 11,087 (33.2%) |
|  |  |  |  |
| Educational attainment | School certificate or lower | 74,336 (32.6%) | 15,139 (46.9%) |
|  | High school, trade, or diploma | 97,884 (42.9%) | 12,170 (37.7%) |
|  | University degree or higher | 55,924 (24.5%) | 4,980 (15.4%) |
|  |  |  |  |
| Residential area | Major city | 103,116 (44.6%) | 15,476 (45.8%) |
|  | Regional/remote | 127,932 (55.4%) | 18,323 (54.2%) |
|  |  |  |  |
| Country of birth | Australia | 174,017 (75.3%) | 24,444 (72.3%) |
|  | Other country | 57,031 (24.7%) | 9,355 (27.7%) |
|  |  |  |  |
| Physician diagnosed cardiovascular or metabolic disease^a^ | No | 175,813 (76.1%) | 24,536 (72.6%) |
|  | Yes | 55,235 (23.9%) | 9,263 (27.4%) |
|  |  |  |  |
| Recent diagnosis of cancer^b^ | No | 212,057 (91.8%) | 31,047 (91.9%) |
|  | Yes | 18,991 (8.2%) | 2,752 (8.1%) |
|  |  |  |  |
| Died during follow-up | Yes | 15,635 (6.8%) | 4,450 (13.2%) |
|  | No | 215,413 (93.2%) | 29,349 (86.8%) |

^a^Cardiovascular or metabolic disease was based on physician diagnosed thrombosis, diabetes, heart disease, stroke or recent treatment (in the last month) for thrombosis, myocardial infarction, or any other type of heart disease.

^b^Recent diagnosis of cancer was based on cancer diagnosis (except for non-melanoma skin cancer) within the past 10 years prior to the baseline data collection
