# Supplementary material for: Traditional and Emerging Lifestyle Risk Behaviors and All-Cause Mortality in Middle-Aged and Older Adults: Evidence from a Large Population-Based Australian Cohort
Source: PLoS Med. 2015 Dec 8;12(12):e1001917. doi: 10.1371/journal.pmed.1001917 (PMC4672919; doi:10.1371/journal.pmed.1001917)
Supplement: S2 Table — (DOC) [file pmed.1001917.s002.doc]

**S2 Table. Sensitivity analysis: prevalence of all 96 combinations of lifestyle risk behaviors and adjusted hazard ratios for their associations with all-cause mortality after excluding deaths within the first two years of follow-up(2006-2014, n=227,346)**

| **Lifestyle risk behavior** | | | | | | |  |  |
| --- | --- | --- | --- | --- | --- | --- | --- | --- |
| **Smoking** | **Poor Diet** | **High Alcohol Intake** | **Physical inactivity** | **Prolonged sitting** | **Long Sleep Duration** | **Short Sleep Duration** | **Percent** | **HR^a^** |
| 0 | 0 | 0 | 0 | 0 | 0 | 0 | 31.43 | Reference |
| 0 | 0 | 0 | 0 | 1 | 0 | 0 | 9.21 | 1.16 (1.06 - 1.26) |
| 0 | 0 | 0 | 1 | 0 | 0 | 0 | 7.11 | 1.57 (1.47 - 1.69) |
| 0 | 1 | 0 | 0 | 0 | 0 | 0 | 6.95 | 1.09 (0.99 - 1.19) |
| 0 | 0 | 0 | 0 | 0 | 0 | 1 | 5.69 | 1.11 (1.01 - 1.21) |
| 0 | 0 | 1 | 0 | 0 | 0 | 0 | 4.19 | 1.06 (0.95 - 1.18) |
| 0 | 0 | 0 | 1 | 1 | 0 | 0 | 2.86 | 2.21 (2.02 - 2.41) |
| 0 | 0 | 0 | 0 | 0 | 1 | 0 | 2.33 | 1.40 (1.26 - 1.55) |
| 0 | 1 | 0 | 0 | 1 | 0 | 0 | 2.26 | 1.09 (0.93 - 1.28) |
| 0 | 0 | 0 | 0 | 1 | 0 | 1 | 1.76 | 1.11 (0.92 - 1.33) |
| 0 | 0 | 0 | 1 | 0 | 0 | 1 | 1.73 | 1.49 (1.32 - 1.68) |
| 0 | 0 | 1 | 1 | 0 | 0 | 0 | 1.59 | 1.76 (1.56 - 1.98) |
| 0 | 1 | 1 | 0 | 0 | 0 | 0 | 1.56 | 1.23 (1.05 - 1.44) |
| 0 | 0 | 1 | 0 | 1 | 0 | 0 | 1.48 | 1.33 (1.12 - 1.58) |
| 1 | 0 | 0 | 0 | 0 | 0 | 0 | 1.40 | 2.00 (1.66 - 2.42) |
| 0 | 1 | 0 | 1 | 0 | 0 | 0 | 1.14 | 1.50 (1.29 - 1.74) |
| 0 | 0 | 0 | 1 | 0 | 1 | 0 | 1.04 | 2.31 (2.08 - 2.57) |
| 0 | 0 | 1 | 0 | 0 | 0 | 1 | 0.93 | 1.27 (1.04 - 1.54) |
| 0 | 1 | 0 | 0 | 0 | 0 | 1 | 0.92 | 1.10 (0.90 - 1.35) |
| 0 | 0 | 1 | 1 | 1 | 0 | 0 | 0.73 | 2.22 (1.89 - 2.61) |
| 0 | 0 | 0 | 1 | 1 | 0 | 1 | 0.71 | 2.31 (1.97 - 2.71) |
| 0 | 1 | 0 | 0 | 0 | 1 | 0 | 0.68 | 1.42 (1.19 - 1.68) |
| 1 | 0 | 1 | 0 | 0 | 0 | 0 | 0.63 | 2.98 (2.39 - 3.70) |
| 0 | 1 | 1 | 0 | 1 | 0 | 0 | 0.55 | 1.00 (0.72 - 1.38) |
| 1 | 1 | 0 | 0 | 0 | 0 | 0 | 0.52 | 2.28 (1.73 - 3.01) |
| 0 | 1 | 0 | 1 | 1 | 0 | 0 | 0.51 | 1.87 (1.50 - 2.32) |
| 0 | 0 | 0 | 1 | 1 | 1 | 0 | 0.49 | 3.62 (3.23 - 4.06) |
| 0 | 0 | 0 | 0 | 1 | 1 | 0 | 0.49 | 1.68 (1.41 - 2.01) |
| 0 | 0 | 1 | 0 | 0 | 1 | 0 | 0.42 | 1.49 (1.22 - 1.82) |
| 1 | 0 | 0 | 0 | 0 | 0 | 1 | 0.42 | 2.89 (2.14 - 3.89) |
| 0 | 0 | 1 | 1 | 0 | 0 | 1 | 0.41 | 1.98 (1.61 - 2.42) |
| 0 | 1 | 1 | 1 | 0 | 0 | 0 | 0.40 | 1.59 (1.23 - 2.05) |
| 1 | 0 | 0 | 1 | 0 | 0 | 0 | 0.40 | 1.96 (1.45 - 2.65) |
| 1 | 0 | 0 | 0 | 1 | 0 | 0 | 0.38 | 2.68 (1.94 - 3.71) |
| 0 | 1 | 0 | 0 | 1 | 0 | 1 | 0.38 | 1.10 (0.75 - 1.60) |
| 0 | 0 | 1 | 0 | 1 | 0 | 1 | 0.35 | 1.58 (1.13 - 2.19) |
| 1 | 1 | 1 | 0 | 0 | 0 | 0 | 0.35 | 2.02 (1.46 - 2.80) |
| 0 | 0 | 1 | 1 | 0 | 1 | 0 | 0.29 | 2.45 (2.07 - 2.91) |
| 1 | 0 | 1 | 1 | 0 | 0 | 0 | 0.25 | 2.60 (1.83 - 3.69) |
| 0 | 1 | 1 | 0 | 0 | 0 | 1 | 0.25 | 1.20 (0.82 - 1.75) |
| 1 | 0 | 1 | 0 | 0 | 0 | 1 | 0.22 | 5.10 (3.68 - 7.06) |
| 0 | 1 | 0 | 1 | 0 | 0 | 1 | 0.21 | 1.37 (0.97 - 1.93) |
| 0 | 1 | 1 | 0 | 0 | 1 | 0 | 0.21 | 1.17 (0.84 - 1.64) |
| 0 | 1 | 0 | 1 | 0 | 1 | 0 | 0.20 | 2.17 (1.73 - 2.73) |
| 0 | 0 | 1 | 1 | 1 | 0 | 1 | 0.20 | 2.08 (1.56 - 2.79) |
| 0 | 1 | 1 | 1 | 1 | 0 | 0 | 0.20 | 2.08 (1.48 - 2.92) |
| 1 | 0 | 1 | 0 | 1 | 0 | 0 | 0.19 | 3.48 (2.38 - 5.09) |
| 1 | 0 | 0 | 1 | 1 | 0 | 0 | 0.17 | 2.82 (1.94 - 4.09) |
| 1 | 1 | 0 | 0 | 1 | 0 | 0 | 0.15 | 2.16 (1.28 - 3.66) |
| 0 | 0 | 1 | 1 | 1 | 1 | 0 | 0.15 | 3.35 (2.75 - 4.07) |
| 0 | 1 | 0 | 0 | 1 | 1 | 0 | 0.14 | 2.04 (1.50 - 2.77) |
| 1 | 0 | 1 | 1 | 1 | 0 | 0 | 0.14 | 4.66 (3.23 - 6.73) |
| 1 | 0 | 0 | 1 | 0 | 0 | 1 | 0.13 | 3.38 (2.17 - 5.25) |
| 1 | 1 | 0 | 1 | 0 | 0 | 0 | 0.12 | 2.63 (1.63 - 4.24) |
| 1 | 0 | 0 | 0 | 1 | 0 | 1 | 0.11 | 3.84 (2.31 - 6.39) |
| 1 | 0 | 0 | 0 | 0 | 1 | 0 | 0.11 | 1.93 (1.20 - 3.10) |
| 0 | 0 | 1 | 0 | 1 | 1 | 0 | 0.11 | 1.71 (1.21 - 2.43) |
| 1 | 1 | 1 | 0 | 1 | 0 | 0 | 0.11 | 3.16 (1.87 - 5.36) |
| 0 | 1 | 0 | 1 | 1 | 0 | 1 | 0.10 | 3.26 (2.16 - 4.91) |
| 1 | 0 | 1 | 1 | 0 | 0 | 1 | 0.10 | 4.20 (2.64 - 6.67) |
| 1 | 1 | 1 | 1 | 0 | 0 | 0 | 0.10 | 1.94 (1.04 - 3.61) |
| 0 | 1 | 1 | 0 | 1 | 0 | 1 | 0.10 | 1.46 (0.73 - 2.93) |
| 0 | 1 | 1 | 1 | 0 | 0 | 1 | 0.10 | 1.85 (1.11 - 3.07) |
| 1 | 1 | 0 | 0 | 0 | 0 | 1 | 0.10 | 3.03 (1.67 - 5.48) |
| 1 | 0 | 1 | 0 | 1 | 0 | 1 | 0.09 | 3.84 (2.12 - 6.95) |
| 0 | 1 | 1 | 1 | 0 | 1 | 0 | 0.08 | 1.67 (1.15 - 2.45) |
| 1 | 1 | 1 | 0 | 0 | 0 | 1 | 0.08 | 2.56 (1.33 - 4.94) |
| 1 | 0 | 1 | 0 | 0 | 1 | 0 | 0.08 | 3.74 (2.32 - 6.03) |
| 0 | 1 | 0 | 1 | 1 | 1 | 0 | 0.07 | 2.82 (2.06 - 3.86) |
| 1 | 0 | 0 | 1 | 1 | 0 | 1 | 0.06 | 4.06 (2.35 - 7.00) |
| 1 | 1 | 0 | 1 | 1 | 0 | 0 | 0.06 | 2.63 (1.25 - 5.53) |
| 1 | 0 | 0 | 1 | 0 | 1 | 0 | 0.06 | 4.73 (3.16 - 7.08) |
| 1 | 0 | 1 | 1 | 0 | 1 | 0 | 0.06 | 2.76 (1.48 - 5.14) |
| 1 | 0 | 1 | 1 | 1 | 0 | 1 | 0.05 | 5.04 (2.92 - 8.70) |
| 1 | 1 | 1 | 0 | 0 | 1 | 0 | 0.05 | 3.88 (2.15 - 7.02) |
| 1 | 1 | 1 | 1 | 1 | 0 | 0 | 0.05 | 5.81 (3.49 - 9.66) |
| 1 | 1 | 0 | 0 | 0 | 1 | 0 | 0.05 | 3.41 (1.93 - 6.01) |
| 0 | 1 | 1 | 1 | 1 | 0 | 1 | 0.04 | 1.87 (0.93 - 3.74) |
| 0 | 1 | 1 | 0 | 1 | 1 | 0 | 0.04 | 2.15 (1.31 - 3.52) |
| 1 | 1 | 0 | 0 | 1 | 0 | 1 | 0.04 | 2.64 (0.99 - 7.05) |
| 0 | 1 | 1 | 1 | 1 | 1 | 0 | 0.04 | 2.54 (1.53 - 4.23) |
| 1 | 0 | 0 | 0 | 1 | 1 | 0 | 0.03 | 3.56 (1.60 - 7.94) |
| 1 | 0 | 0 | 1 | 1 | 1 | 0 | 0.03 | 8.14 (5.18 - 12.79) |
| 1 | 0 | 1 | 1 | 1 | 1 | 0 | 0.03 | 9.44 (6.07 - 14.66) |
| 1 | 1 | 1 | 1 | 0 | 1 | 0 | 0.03 | 3.11 (1.29 - 7.47) |
| 1 | 1 | 1 | 0 | 1 | 0 | 1 | 0.03 | 6.75 (3.21 - 14.18) |
| 1 | 1 | 1 | 1 | 0 | 0 | 1 | 0.03 | 3.89 (1.62 - 9.37) |
| 1 | 1 | 0 | 1 | 0 | 0 | 1 | 0.03 | 2.53 (0.81 - 7.85) |
| 1 | 1 | 0 | 1 | 0 | 1 | 0 | 0.03 | 2.44 (1.02 - 5.88) |
| 1 | 1 | 1 | 1 | 1 | 0 | 1 | 0.02 | 4.34 (2.07 - 9.13) |
| 1 | 0 | 1 | 0 | 1 | 1 | 0 | 0.02 | 4.76 (2.26 - 10.00) |
| 1 | 1 | 0 | 1 | 1 | 0 | 1 | 0.02 | 6.78 (2.18 - 21.04) |
| 1 | 1 | 1 | 0 | 1 | 1 | 0 | 0.01 | 3.51 (1.31 - 9.35) |
| 1 | 1 | 1 | 1 | 1 | 1 | 0 | 0.01 | 6.83 (3.41 - 13.68) |
| 1 | 1 | 0 | 0 | 1 | 1 | 0 | 0.01 | 2.42 (0.78 - 7.50) |
| 1 | 1 | 0 | 1 | 1 | 1 | 0 | 0.01 | 4.43 (1.11 - 17.75) |

A”1” denotes the presence of a risk behavior and a “0” denotes the absence of a risk behavior

^a^Adjusted for age, sex, educational attainment, marital status, residential area, and country of birth
