# Supplementary material for: Traditional and Emerging Lifestyle Risk Behaviors and All-Cause Mortality in Middle-Aged and Older Adults: Evidence from a Large Population-Based Australian Cohort
Source: PLoS Med. 2015 Dec 8;12(12):e1001917. doi: 10.1371/journal.pmed.1001917 (PMC4672919; doi:10.1371/journal.pmed.1001917)
Supplement: S1 Text — (DOCX) [file pmed.1001917.s003.docx]

**S1 Text - STROBE Statement**

|  | Item No | Recommendation |
| --- | --- | --- |
| **Title and abstract** | 1 | (*a*) Indicate the study’s design with a commonly used term in the title or the abstract – *In both the title and the abstract we explicitly mentioned it is a cohort study.* |
|  |  | (*b*) Provide in the abstract an informative and balanced summary of what was done and what was found – *Clearly stated in abstract.* |
| Introduction | | |
| Background/rationale | 2 | Explain the scientific background and rationale for the investigation being reported –  *-- Clearly stated in the Introduction* |
| Objectives | 3 | State specific objectives, including any prespecified hypotheses  – *Two objectives stated* |
| Methods | | |
| Study design | 4 | Present key elements of study design early in the paper –– METHODS  *-- Stated at the start of Methods* |
| Setting | 5 | Describe the setting, locations, and relevant dates, including periods of recruitment, exposure, follow-up, and data collection – *Stated in Methods* |
| Participants | 6 | (*a*) Give the eligibility criteria, and the sources and methods of selection of participants. Describe methods of follow-up – *eligibility criteria, sources and methods of selection* *were stated in Sampling and Procedures; Methods of follow-up stated in Measures* |
|  |  | (*b*) For matched studies, give matching criteria and number of exposed and unexposed – *NA* |
| Variables | 7 | Clearly define all outcomes, exposures, predictors, potential confounders, and effect modifiers – *Clearly defined in Methods* *and Table 1*  Give diagnostic criteria, if applicable – *NA* |
| Data sources/ measurement | 8* | For each variable of interest, give sources of data and details of methods of assessment (measurement) – *References for measures provided whenever possible*  Describe comparability of assessment methods if there is more than one group – *NA* |
| Bias | 9 | Describe any efforts to address potential sources of bias – *Sensitivity analysis conducted as stated in Statistical Analysis* |
| Study size | 10 | Explain how the study size was arrived at – *Stated in Results and presented in Figure 1* |
| Quantitative variables | 11 | Explain how quantitative variables were handled in the analyses. If applicable, describe which groupings were chosen and why – *Stated in Methods (Measures and Statistical Analysis)* |
| Statistical methods | 12 | (*a*) Describe all statistical methods, including those used to control for confounding – *Stated in Methods (Statistical Analysis)* |
|  |  | (*b*) Describe any methods used to examine subgroups and interactions – *Stated in Methods (Statistical Analysis)* |
|  |  | (*c*) Explain how missing data were addressed – *Discussed in Methods (Measures and Statistical Analysis)* |
|  |  | (*d*) If applicable, explain how loss to follow-up was addressed – *Loss to follow-up accounted for less than 2% of the total participants* |
|  |  | (*e*) Describe any sensitivity analyses – *Sensitivity analysis conducted as stated in Statistical Analysis and Results* |
| Results | | |
| Participants | 13* | (a) Report numbers of individuals at each stage of study—eg numbers potentially eligible, examined for eligibility , confirmed eligible , included in the study, completing follow-up , and analysed – *Presented in Figure 1* |
|  |  | (b) Give reasons for non-participation at each stage –*Presented in Figure 1* |
|  |  | (c) Consider use of a flow diagram – *Figure 1* |
| Descriptive data | 14* | (a) Give characteristics of study participants (eg demographic, clinical, social) and information on exposures and potential confounders – *Stated in Results (Descriptive Statistics) and Table 2* |
|  |  | (b) Indicate number of participants with missing data for each variable of interest – *Indicated in Table 2* |
|  |  | (c) Summarise follow-up time (eg, average and total amount) – *Indicated in Results (Descriptive Statistics)* |
| Outcome data | 15* | Report numbers of outcome events or summary measures over time – *Indicated in Results and Table 3* |
| Main results | 16 | (*a*) Give unadjusted estimates and, if applicable, confounder-adjusted estimates and their precision (eg, 95% confidence interval). Make clear which confounders were adjusted for and why they were included – *Indicated in Results , Table 3 and 4* |
|  |  | (*b*) Report category boundaries when continuous variables were categorized – *Indicated in Table 1, 2, and 3* |
|  |  | (*c*) If relevant, consider translating estimates of relative risk into absolute risk for a meaningful time period – *We have presented both cumulative death rates and hazard ratios in Table 3* |
| Other analyses | 17 | Report other analyses done—eg analyses of subgroups and interactions, and sensitivity analyses – *Sensitivity and subgroup analysis presented in Results and Table 3* |
| Discussion | | |
| Key results | 18 | Summarise key results with reference to study objectives – *Addressed* |
| Limitations | 19 | Discuss limitations of the study, taking into account sources of potential bias or imprecision. Discuss both direction and magnitude of any potential bias – *Extensively described in Discussion (Strengths and Limitations)* |
| Interpretation | 20 | Give a cautious overall interpretation of results considering objectives, limitations, multiplicity of analyses, results from similar studies, and other relevant evidence – *Cautious interpretation provided in Discussion* |
| Generalisability | 21 | Discuss the generalisability (external validity) of the study results – *Addressed in Discussion* |
| Other information | | |
| Funding | 22 | Give the source of funding and the role of the funders for the present study and, if applicable, for the original study on which the present article is based – *Funding and the role of the funder presented.* |

*Give information separately for exposed and unexposed groups.

**Note:** An Explanation and Elaboration article discusses each checklist item and gives methodological background and published examples of transparent reporting. The STROBE checklist is best used in conjunction with this article (freely available on the Web sites of PLoS Medicine at http://www.plosmedicine.org/, Annals of Internal Medicine at http://www.annals.org/, and Epidemiology at http://www.epidem.com/). Information on the STROBE Initiative is available at http://www.strobe-statement.org.
