# Supplementary material for: Traditional and Emerging Lifestyle Risk Behaviors and All-Cause Mortality in Middle-Aged and Older Adults: Evidence from a Large Population-Based Australian Cohort
Source: PLoS Med. 2015 Dec 8;12(12):e1001917. doi: 10.1371/journal.pmed.1001917 (PMC4672919; doi:10.1371/journal.pmed.1001917)
Supplement: S2 Text — (DOC) [file pmed.1001917.s004.doc]

**Traditional and emerging lifestyle risk behaviors and all-cause mortality in middle-aged and older adults: Evidence from a large population-based Australian cohort**

**Data analysis plans**

**Overall statement**

This study aims to 1) examine the association between a lifestyle risk index and all-cause mortality and to quantify the population attributable risk associated with the risk score, and 2) to describe the most commonly occurring combinations of health risk behaviors *and to quantify the risk for all-cause mortality for each unique lifestyle combination**. Using the 45 and Up data, we will create a summary lifestyle index, as a score including physical inactivity, poor diet, smoking, alcohol, sedentary behavior, and sleep. In addition to creating a lifestyle risk index, we plan to explore different combinations of risk factors in relation to all-cause mortality, which is based on the most updated mortality linkage.

**Specific analysis plans**

***Exposure variables:***

|  | **Two categories** | |
| --- | --- | --- |
| **Risks** | **0** | **1** |
| **Smoking** | Never/past smoker | Current smoker |
| **Physical Activity** | ≥300 mins, 150-299 | <150 mins |
| **Alcohol** | ≤ 14 drinks/week | >14 drinks/week |
| **Diet** | Score 6-10 | Score 0-5 |
| **Sleep** | 7-9 hours/day | <7 or >9 hours/day |
| **Sitting** | ≤ 7 hours/day | >7 hours/day |

***Outcome variable:*** All-cause mortality from Registry of Births, Deaths, and Marriages (RBDM) administrative data (check and exclude duplicated records first)

***Exclusion:***

1. Missing/logically incorrect recruitment date
2. Unmatched cases between the 45 and Up and RBDM data
3. Participants with missing (exposure) lifestyle index
4. Participants with missing areas of residence

Sample sizes will be reported at each stage of exclusion and participant flowchart will be reported.

***Data analysis:***

1. Descriptive statistics: Demographic and health characteristics (i.e., age group, sex, marital status, educational attainment, residential area, country of birth, physician diagnosed cancer, cardiovascular and metabolic disease) by lifestyle risk index score. Analytical Table 1: Row and column % reported
2. Cox proportional hazards analysis (need to test the proportional hazard assumption prior to each analysis).

Repeat models for the following exposure variables:

1. Each individual lifestyle risk behavior (adjusted for covariates and all other risk factors in the same model)---report in text
2. Lifestyle risk index score --- report in Analytical Table 2 (c-index and PAR report in text)
3. Combinations of lifestyle risk behaviors (top common 15 combinations prior to first draft*, all combinations for revision and resubmission**; 64 total combinations for the initial draft, *96 (sleep split into three categories, <7, 7-9,>9) for revision and resubmission)* --- Analytical Table 3
4. Test for effect modification and subgroup analysis:

Age group, sex, educational attainment, diagnosis with cardiovascular and metabolic disease, recent cancer diagnosis (last 10 years, all cancers except non-melanoma skin cancer), *BMI categories (added for revision and resubmission*)*

1. Sensitivity analysis:
2. Repeat analysis excluding deaths within the first 2 years
3. *Repeat analysis adjusted for major diseases (cardiovascular disease and recent cancer; and the chronic disease index)*
4. *Repeat analysis adjusted for BMI categories*

**Covariates:** age categories (10 years per category), sex, educational attainment (≤school certificate; high school, trade or diploma; ≥university degree), marital status (not in a cohabitating relationship vs. in a cohabitating relationship), country of birth (Australia vs. other countries) and Accessibility/Remoteness Index of Australia (classified as major city, inner and regional, outer regional, remote)

**Missing data handling:**

- Missing key exposure measures (any risk behaviors)---exclude without imputation
- Missing covariates --- included in the analysis with missing as a category

**new analysis added after the first round of peer-review.*
